# Impact of Synaptic Device Variations on Pattern Recognition Accuracy in a Hardware Neural Network

**DOI:** 10.1038/s41598-018-21057-x

**Published:** 2018-02-08

**Authors:** Sungho Kim, Meehyun Lim, Yeamin Kim, Hee-Dong Kim, Sung-Jin Choi

**Affiliations:** 1 Department of Electrical Engineering, Sejong University, Seoul, 05006 Korea; 2 Mechatronics R&D Center, Samsung Electronics, Gyonggi-do, 18448 Korea; 3 School of Electrical Engineering, Kookmin University, Seoul, 02707 Korea

## Abstract

Neuromorphic systems (hardware neural networks) derive inspiration from biological neural systems and are expected to be a computing breakthrough beyond conventional von Neumann architecture. Interestingly, in neuromorphic systems, the processing and storing of information can be performed simultaneously by modulating the connection strength of a synaptic device (*i*.*e*., synaptic weight). Previously investigated synaptic devices can emulate the functionality of biological synapses successfully by utilizing various nano-electronic phenomena; however, the impact of intrinsic synaptic device variability on the system performance has not yet been studied. Here, we perform a device-to-system level simulation of different synaptic device variation parameters in a designed neuromorphic system that has the potential for unsupervised learning and pattern recognition. The effects of variations in parameters such as the weight modulation nonlinearity (*NL*), the minimum-maximum weight (*G*_*min*_ and *G*_*max*_), and the weight update margin (*ΔG*) on the pattern recognition accuracy are analyzed quantitatively. These simulation results can provide guidelines for the continued design and optimization of a synaptic device for realizing a functional large-scale neuromorphic computing system.

## Introduction

The mammalian neocortex offers extremely energy-efficient information processing performance in tasks such as pattern recognition with a power consumption of only 10–20 watts^1^. By mimicking both the functional and structural advantages of this biological neural system, the recent development of power-efficient computing systems, *i*.*e*., neuromorphic systems (hardware neural networks)^2^, has been expected to offer a promising breakthrough for applications, ranging from mobile platforms^3^ to artificial intelligence operations^4^, where power consumption is a concern.

A unique feature of neuromorphic systems is efficient parallel data processing, where the processing of information can be performed by modulating the connection strength of synapses (referred to as the synaptic weight)^5^. This synaptic weight can be modulated by either potentiating or depressing neural spikes (pulses) from pre- and post-synaptic neurons, following appropriate learning rules, such as spike-timing-dependent plasticity (STDP)^6^. Therefore, a key element in the neuromorphic system is the implementation of an ideal synaptic device that can emulate the functionality of biological synapses.

To date, various nano-electronic devices have successfully reproduced a specific learning rule of biological synapses through their internal analog conductance states that can be modulated intentionally with an applied pulse’s timing or level^7–15^. Moreover, the potential of ultralow energy consumption per synaptic operation^16^, as well as the possibility of realizing three-dimensional integration^17^, have shown the promising feasibility of large-scale neuromorphic system implementation in the near future. However, the sustainability of such devices is still in doubt due to the variability issue that is common to all nano-electronic devices^18–20^. The physical mechanism of the conductance modulation in most prospective synaptic devices, which is typically a random process in an atomic-level change based on electro/thermo-dynamics^21^, is responsible for an unavoidable device-to-device^22^ variation. In particular, the effect of device variations on the neuromorphic system performance, *e*.*g*. pattern recognition accuracy, has not been analyzed quantitatively^23–28^, which leads to a bottleneck in the continuing design and optimization of synaptic devices and the entire system.

In this study, a device-to-system level simulation presents quantitative results in terms of the neuromorphic system performance depending on different device variation parameters, where the discussed system has the potential for unsupervised online learning and consequent image classification for MNIST handwritten digits. The effect of the synaptic device variations in the weight modulation nonlinearity (*NL*), the minimum-maximum weight (*G*_*min*_ and *G*_*max*_), and the weight update margin (*ΔG*) on the pattern recognition accuracy is analyzed. These simulation results provide a design guideline for the specifications of the synaptic device needed for more reliable system operation. Here, note that our analysis is only limited to the neuromorphic system that are capable of online learning. Since the system operated by offline-learning is consisted of only synaptic device network and dos not require peripheral circuits, the effects of different device variation parameters to the system performance is easily predictable and well-discussed by previous studies^23–25^. In contrast, the comprehensive study for the effects of device variation to the system performance with online-learning has been missing^29–32^, thus our study aims the quantitative analysis to understand how the pattern recognition accuracy of the existing STDP-based online-learning system affected by device variation parameters.

## Results and Discussion

**Figure 1:**
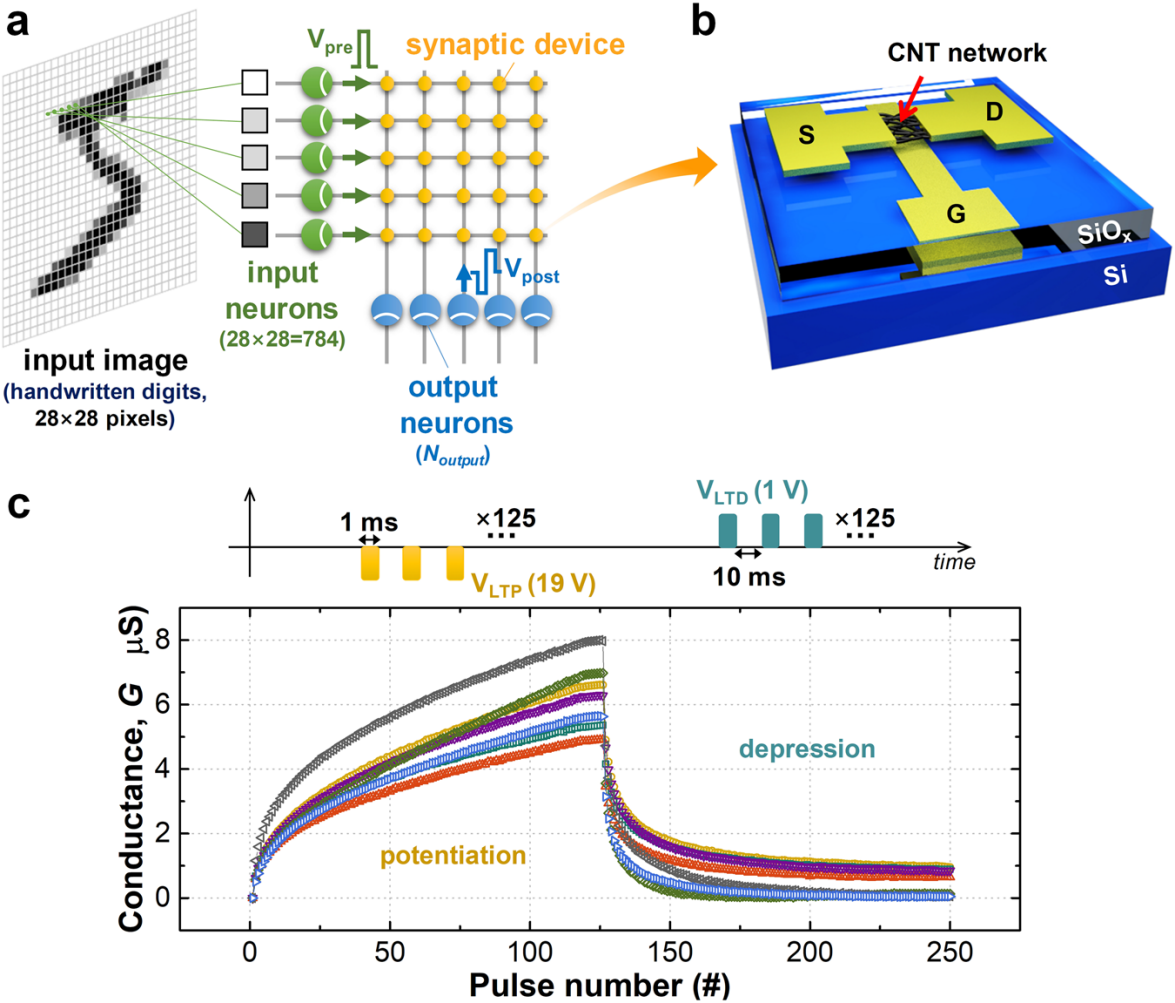
(**a**) The synaptic device network for pattern recognition of 28 × 28 grayscale images consisting of the input and output layers. The input neuron is fully connected to the input image pixel in a one-to-one manner. The synaptic devices are located at the junctions between the input and output neurons. (**b**) The fabricated synaptic device; the CNT-based synaptic transistor emulates the functionalities of biological synapse through the analog channel conductance modulation. (**c**) The example of the device-to-device variation of our synaptic devices. The measured analog conductance modulation characteristics are from different 7 devices.

In our previous work, we demonstrated a device-to-system level simulation framework along with a designed learning rule^29,30^. Briefly, Fig. 1a shows schematics of the demonstrated system and the learning rule for our conceived pattern recognition task. The detail simulation procedure in our pattern recognition system is presented in Supplementary Information Section 1. With the crossbar layout, each input neuron is connected to one pixel of the image; input neurons emit pre-synaptic pulses (V_pre_) wherein the timing of the pre-synaptic pulses represents the analog information of the pixel intensities. Subsequently, pre-synaptic pulses from the input neurons can trigger multiple synaptic transistors simultaneously, and post-synaptic currents determined by the channel conductance of each synaptic transistor are collected and accumulated at an output neuron. If the accumulated post-synaptic current level is greater than a given threshold value, one output neuron fires a post-synaptic pulse (V_post_); then, the synaptic weight can be modulated to any analog state according to the correlation of the pre- and post-synaptic pulses. Here, the synaptic device based on the carbon nanotube (CNT) transistor^29,30^ has demonstrated the functionality of synaptic weight as shown in Fig. 1b. The characteristic of analog channel conductance modulation in this synaptic device is input to the simulation as a parameter, and consequently, the system-level pattern recognition procedure can be simulated in regard to the synaptic device characteristic, *i*.*e*., a device-to-system level simulation framework. However, our previous study had a critical limitation because the effect of synaptic device variations was ignored. Despite the intrinsic device-to-device variation of the synaptic devices as shown in Fig. 1c, the simulation was performed by reflecting only one device characteristic and assuming all synaptic devices had the equivalent characteristic^29,30^ (the discussions about the origin of variation and working principle in our synaptic device are presented in the Supplementary Information Section 2 and 3, respectively). Since this approach has been common in other studies for simulation simplicity^31–33^, an accurate quantitative analysis of such a neuromorphic system has not yet been conducted. Therefore, in this study, we investigate how different synaptic device variations affect the pattern recognition accuracy of the system by using our simulation and considering the device-to-device variation.

**Figure 2:**
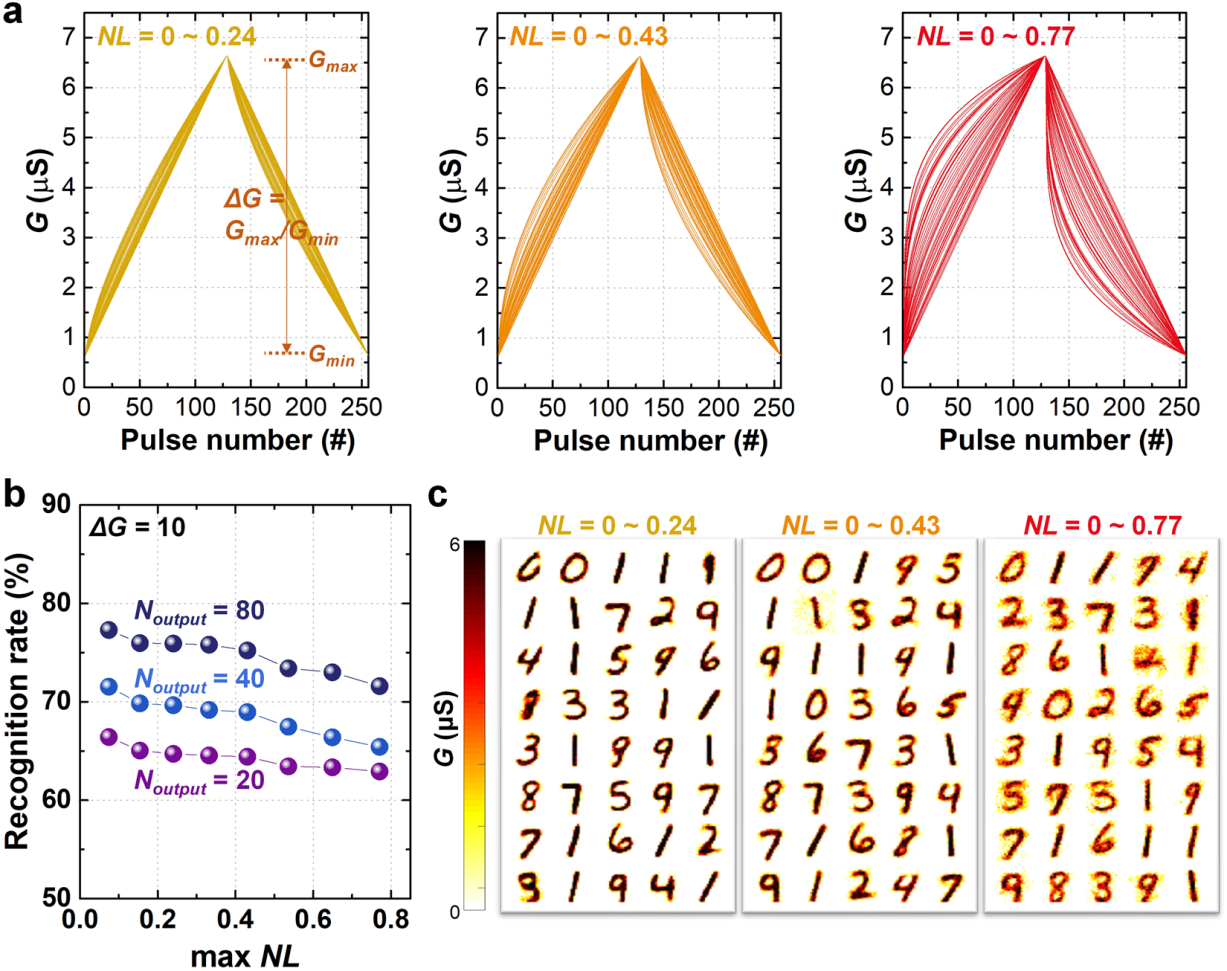
(**a**) The synaptic device conductance (*G*) as a function of applied pulse number with randomly assigned *NL* values. Actually, the total number of synaptic devices is 28 × 28 × *N*_*output*_(40) = 31360. In this plot, we plot 50 selected curves only for clarity. (**b**) The simulated recognition rate as a function of maximum *NL* value after 60000 times of training epochs (*ΔG* is fixed at 10). (**c**) The synaptic weights between the input to output neurons with 40 output neurons, when *NL* ranges are 0 ~ 0.24 and 0 ~ 0.77.

First, we investigate the impact of the weight modulation nonlinearity (*NL*) variation on the recognition accuracy of the system. In general, the conductance of the synaptic device (*G*) is more dramatically changed during the first few potentiation/depression pulses and becomes saturated as the number of pulses increases. In other words, every pulse results in a different response in the weight modulation depending on the current weight state, and the cumulative effect on the weight modulation does not follow a simple linear relation, which is attributed to the *NL* of the weight modulation. *NL* can be defined quantitatively as

1 $$NL=Max|{G}_{P}(n)-{G}_{D}(n)|\quad \quad {\rm{for}}\,n=1\,{\rm{to}}\,N{,}$$

where *G*_*P*_(n) and *G*_*D*_(n) are the conductance (weight) values after the *n*th potentiation pulse and *n*th depression pulse, respectively (the *NL* value is normalized to the total conductance change during an update sequence comprising an equal number (*N*) of consecutive potentiating/depressing pulses). Ideally, *NL* should be zero for a completely linear conductance modulation, but *NL* is always greater than zero in the typical synaptic devices. To identify the effect of *NL* on the recognition accuracy, different *NL* values are randomly assigned to each synaptic device (with uniform distribution), and the simulation is performed on the system with different *NL* ranges as shown in Fig. 2a, *e*.*g*., *NL* values of the synaptic devices range from 0 ~ 0.24, 0 ~ 0.4, and 0 ~ 0.77. Figure 2b shows the recognition rate (*i*.*e*., classification accuracy) for the test images as a function of *NL* range, and Fig. 2c shows the trained images (*i*.*e*., the maps of synaptic weight associated with each output neuron) at the system with different *NL* range. Obviously, the recognition rate is degraded by increasing *NL* (Fig. 2b). The reason for this degradation is due to the convergence issue of the trained image. As the training process is repeated, the pixel information of the input images is gradually trained (stored) as the analog conductance value of the synaptic devices. If the *NL* value is increased, the conductance change of the synaptic device occurs abruptly, causing difficulty in the convergence of the conductance into a final stable value. Consequently, irregular pixel values (noise-like) occur in the background of the trained image as shown in Fig. 2c, which results in the degradation of the recognition rate. However, it should be noted that the effect of *NL* is not critical in maintaining the recognition rate; the recognition rate reduction by *NL* is only 6% from the best to the worst cases (when *N*_*output*_ = 40, Fig. 2b). Therefore, it can be concluded that the nonlinearity of the weight modulation at the synaptic device, *i*.*e*., *NL*, is not critical to the recognition rate of the system. This implies that further synaptic device study should be devoted more to improving characteristics other than *NL*, unlike other previous efforts to improve *NL*^24,34^.

Note that this conclusion is the opposite of the results in previous studies, where increasing *NL* degrade the recognition rate obviously^24,34^. For example, in previous work based on conventional backpropagation learning rule with neural network architecture^24^, because all synaptic weights (along with the function of the perceptron) in the whole neural network contribute to the learning process, a precise adjustment of all synaptic weights is necessarily required. In contrast, in our system, since only the synaptic weight associated with one fired output neuron involves the learning process, an iterative modulation of the synaptic weight is more important than the precise adjustment. Consequently, this difference indicates that the required specification of the synaptic device is not determinative, but it depends on what learning rules and architecture are used to construct the system. Only studies to reduce *NL* value^34–38^ are not always the best way to optimize the synaptic device performance.

**Figure 3:**
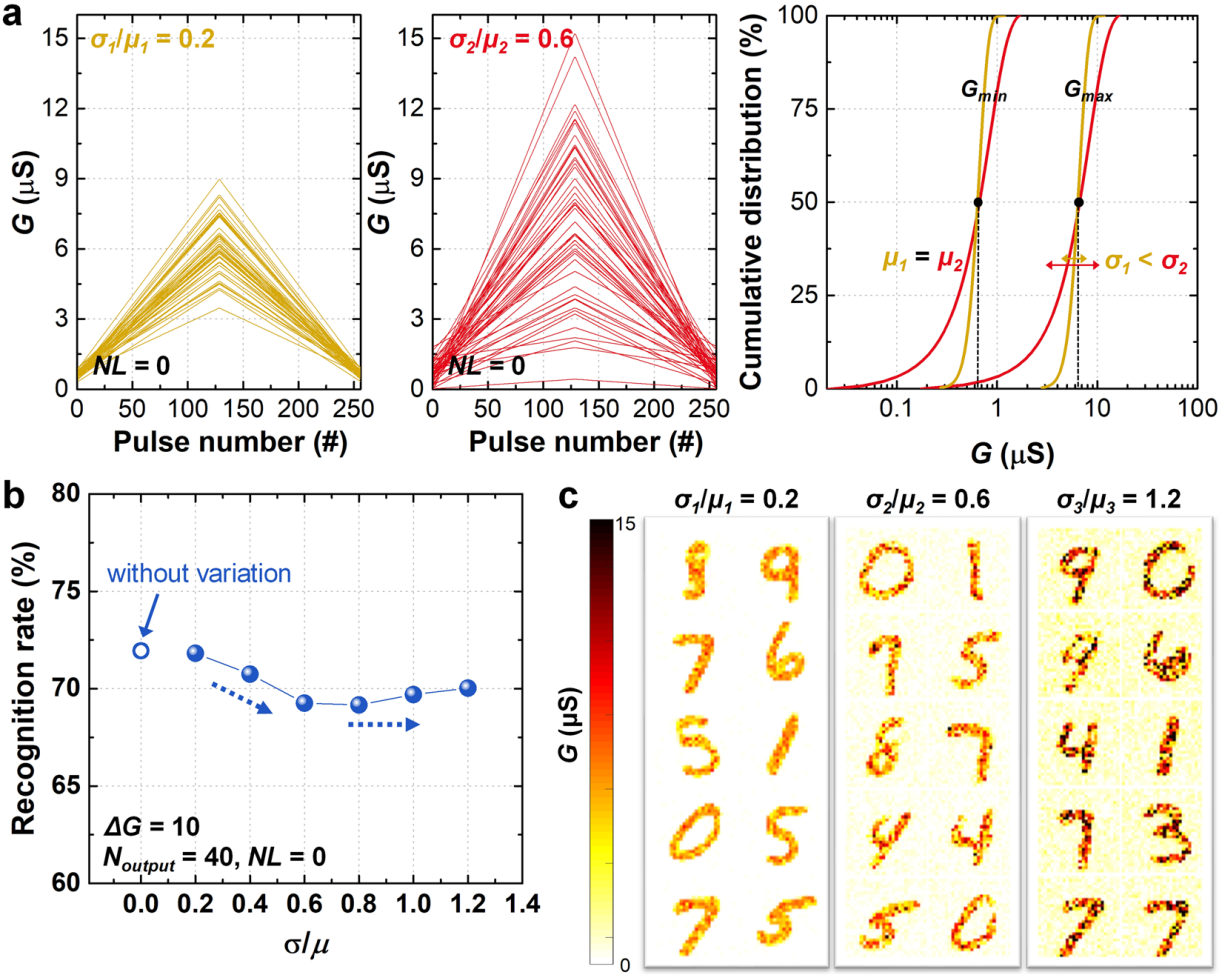
(**a**) The synaptic device conductance (*G*) as a function of applied pulse number with randomly assigned relative standard dispersion *σ*/*μ*. Similar to Fig. 2(a), we plot 50 selected curves only for clarity. The cumulative distribution plot shows the dispersion of the conductance value according to different *σ*/*μ* ratios. (**b**) The simulated recognition rate as a function of the *σ*/*μ* ratio after 60000 times of training epochs (*ΔG* is fixed to 10, *N*_*output*_ is fixed at 40, and *NL* is fixed at 0). (**c**) The synaptic weights between the input to output neurons with 40 output neurons, when *σ*/*μ* is 0.2, 0.6, and 1.2.

A similar analysis was performed to study the effect of the minimum-maximum weight (*G*_*min*_ and *G*_*max*_) variation on the recognition accuracy. With *NL* fixed at zero, Fig. 3a represents different cases of *G*_*min*_ and *G*_*max*_ variations depending on the ratio of the standard deviation (*σ*) to the mean (*μ*). Here, the mean of *G*_*min*_ and *G*_*max*_ is fixed, but the standard deviation is changed; thus, a larger ratio of *σ*/*μ* implies more fluctuation of *G*_*min*_ and *G*_*max*_ values among the synaptic devices. Figure 3b shows the simulated recognition rate as a function of the *σ*/*μ* ratio (with a fixed *ΔG* = 10). Interestingly, the recognition rate decreases as *σ*/*μ* increases, but the recognition rate is maintained above a certain value (*σ*/*μ* = 0.8). The reason for this result can be inferred from the trained image at the synaptic devices, as shown in Fig. 3c. Due to the fluctuation of *G*_*min*_ and *G*_*max*_ among the synaptic devices, the pixel values that converge after the training process is completed fluctuates. Consequently, as *σ*/*μ* increases from 0.2 to 0.6, irregular pixel values (noise) are present in the background of the trained images, which leads to a degradation of the recognition rate. However, as *σ*/*μ* increases further from 0.6 to 1.2, the noise in the background can be compensated by the effect of increasing the pixel value of the main image. In other words, the pixel value difference between the main image and the background becomes larger by increasing *σ*/*μ*, which makes the image more distinct and easier to distinguish. Therefore, the variation of the minimum-maximum weight at the synaptic devices, *i*.*e*., *G*_*min*_ and *G*_*max*_, determines the recognition rate through the correlation of the increase of irregular background noise and the image distinguishability.

**Figure 4:**
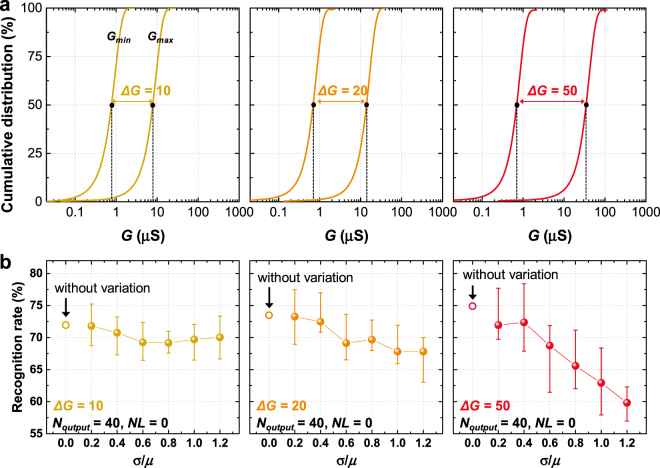
(**a**) Cumulative distribution plots with different weight update margins (*ΔG*) along with the minimum-maximum weight (*G*_*min*_ and *G*_*max*_) variation. (**b**) The simulated recognition rate as a function of the *σ*/*μ* ratio after 60000 times of training epochs (*N*_*output*_ is fixed at 40, and *NL* is fixed at 0).

Finally, the analysis was performed on the effect of the weight update margin (*ΔG*) on the recognition accuracy. The simulation was performed in three different cases (*ΔG* = 10, 20, and 50) along with *σ*/*μ* ratio variation as shown in Fig. 4a and b. Figure 4b shows the simulated the recognition rate as a function of the *σ*/*μ* ratio (Fig. 4b shows repeated simulation results of 20 times since the randomly assigned characteristics of the synaptic devices are used in each simulation time). As shown in Fig. 4b, as discussed above, the variation of *G*_*min*_ and *G*_*max*_ cannot significantly affect the recognition rate when *ΔG* is only 10. However, when *ΔG* increases to 20 and 50, a noticeable degradation in recognition rate is observed. This is because the absolute fluctuation of the weight is increased as *ΔG* is increased under the same standard deviation, leading to significant background noise. Thus, the effect of the increase of irregular background noise overwhelms the effect of the distinguishability increase, which results in a noticeable reduction of the recognition rate.

In summary, we have analyzed the pattern recognition accuracy of the neuromorphic system in the presence of variation in the synaptic devices by using a device-to-system level simulation framework. It is clear that the effect of the nonlinearity of the weight modulation at the synaptic device (*NL*) is not critical to the recognition rate of the system; thus, current research efforts to improve *NL* are not necessary. Instead, the variation from the minimum-maximum weight (*G*_*min*_ and *G*_*max*_) and the weight update margin (*ΔG*) should be improved for higher pattern recognition accuracy, as these variations lead to unwanted background noise in the trained image. Especially, when the synaptic device has a larger *ΔG*, the influence from the variation getting worse; thus, we need to optimize and design the synaptic device specifications carefully when considering the system performance. A larger *ΔG* value of the synaptic device is not always advantageous, and in fact, the synaptic device with a small *ΔG* is more advantageous for overall system performance owing to the immunity of the variation.

One limit of our study is the lack of mathematical proof for the reason we have explained. However, none of previous simulation studies have considered the system performance difference originated by device-to-device variation, where it has been assumed that the performance of the synaptic devices constituting the network are all the same. In contrast, in this study the attempt to analyze the effects of device-to-device variation has conducted systematically. Therefore, the analyzed results presented in this study will be an important step toward realizing functional neuromorphic systems with proper synaptic device development.

## Methods

### Fabrication of carbon nanotube-based synaptic transistors

Carbon nanotube (CNT) synaptic transistors were initially fabricated on highly p-doped rigid silicon substrates with a thermally grown 50-nm-thick SiO_2_ layer. We used the local back-gate structure for efficient local modulation of the channels in the CNT transistors. To form the local back-gate, the palladium (Pd) layer was first deposited and subsequently patterned using evaporation and a lift-off process. Next, a 50-nm-thick SiO_x_ layer, 10-nm-thick Au layer, and 20-nm-thick SiO_x_ layer were deposited sequentially. The thin Au layer served as a floating gate for charge storage. Then, the top surface of the SiO_x_ layer was functionalized with a 0.1 g/mL poly-L-lysine solution to form an amine-terminated layer, which acted as an effective adhesion layer for the deposition of the CNTs. Subsequently, the CNT network channel was formed by immersing the chip into a 0.01 mg/mL 99%-semiconducting CNT solution (*NanoIntegris*, *Inc*.) for several hours followed by a thorough rinse with isopropanol and DI water. Subsequently, the source/drain electrodes consisting of Ti and Pd layers (each 2 nm and 40 nm, respectively) were deposited and patterned using conventional thermal evaporation and a lift-off process, respectively. Finally, additional photolithography and oxygen plasma steps were conducted to remove unwanted electrical paths, which isolated the devices from one another.

## Electronic supplementary material

Supplementary information
