# Supplementary material for: Impact of Synaptic Device Variations on Pattern Recognition Accuracy in a Hardware Neural Network
Source: Sci Rep. 2018 Feb 8;8:2638. doi: 10.1038/s41598-018-21057-x (PMC5805704; doi:10.1038/s41598-018-21057-x)
Supplement: Supplementary file 1 — Supplementary information [file 41598_2018_21057_MOESM1_ESM.pdf]

Supplementary Information for

Impact of Synaptic Device Variations on Pattern Recognition Accuracy in a Hardware Neural Network

Sungho Kim[1A], Meehyun Lim[2A], Yeamin Kim[3A], Hee-Dong Kim[1] & Sung-Jin Choi[3,*]

[1]Department of Electrical Engineering, Sejong University, Seoul 05006, Korea

[2]Mechatronics R&D Center, Samsung Electronics, Gyonggi-do 18448, Korea

[3]School of Electrical Engineering, Kookmin University, Seoul 02707, Korea

*Correspondence to S. J. C. (sjchoiee@kookmin.ac.kr)

1. Pattern recognition simulation procedure

All simulations were performed using C++ code. The code runs on a traditional personal computer, and the simulation time was approximately 90 min per run on an Intel i7-5820K CPU running at 3.3 GHz, where the number of input neurons was 784 and the number of output neurons was 40 (thus, the number of synapses was 784 × 40 = 31,360).

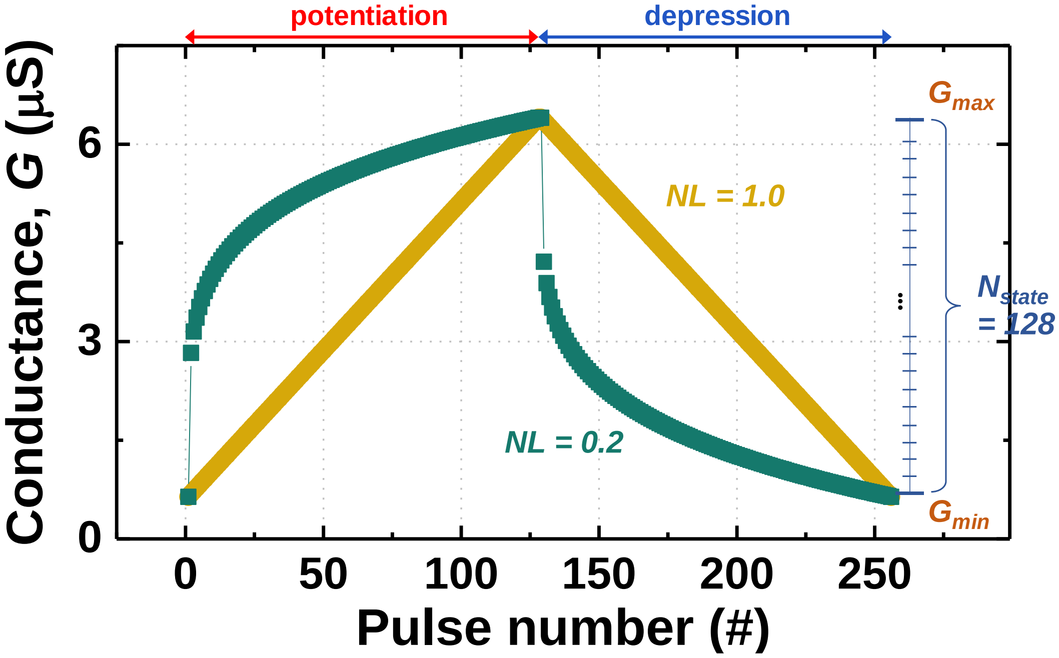

potentiation: $$G(i) = \frac{G_{\max} - G_{\min}}{127^{NL}}(i-1)^{NL} + G_{\min} \qquad i = 1 \text{ to } 128$$

depression: $$G(i) = -G(i-128) + G_{\min} + G_{\max} \qquad i = 129 \text{ to } 256$$

Figure S1. Analog conductance-switching behavior (the data from Fig. 1c) when *NL* = 0.2 and 1.0, respectively.

First, to model the modulations of the synaptic device weight (*G*), an increase (potentiation) and decrease (depression) of *G* was fitted using the following equation:

for potentiation: $$G(i) = \frac{G_{\max} - G_{\min}}{127^{NL}} (i-1)^{NL} + G_{\min} \quad i = 1 \text{ to } 128 \tag{1}$$

for depression: $$G(i) = -G(i-128) + G_{\min} + G_{\max} \quad i = 129 \text{ to } 256 \tag{2}$$

where NL is the weight modulation nonlinearity. Here, for the simplicity of analysis, the number of weight states (N_{state} = 128) and weight update margin ($\Delta G = G_{max}/G_{min}$ = 10) are fixed to 128 and 10, respectively. In addition, G_{min} is defined as 0.64 μS as originated from the real measurement data as shown in Fig. 1c. By assigning random NL, G_{min}, or G_{max} values to each synaptic device, the effect of device-to-device variation can be simulated.

Next, as noted in the main text, the neuron circuit is composed of a leaky integrator, comparator, and waveform generator. A leaky integrator integrates the post-synaptic currents (I_{post}), which is meant to solve the simple following equation:

$$\tau \frac{dX}{dt} + X = I_{post}, \tag{3}$$

where τ = 100 ms. Here, we define the state variable (X) to be an 'integrated' post-synaptic current (I_{post}), and τ is a leak-time constant (the meaning of X is equivalent to the biological membrane potential). The neuron fires a post-synaptic spike if X reaches a given threshold, V_{th}. In addition, when an output neuron fires spikes, it sends inhibitory signals to the other output neurons that prevent them from firing during the specific inhibition time and resets their X values to zero, which is known as the winner-takes-all rule.

Simple algorithms for the homeostasis effect were included in these simulations. A target activity (*i.e.*, the number of times an output neuron should fire within the total simulation time) is defined for the neurons. Then, the threshold of the neuron (V_{th}) is increased if the average activity of the neuron is greater than the target and decreased if it is less than the target:

$$\frac{dV_{th}}{dt} = \gamma(A - T) + \kappa, \tag{4}$$

where A is the mean firing rate of a neuron, T is the target activity, and γ (= 0.5) are κ (= 0.005) are constants. Accordingly, all thresholds of the output neurons are adjusted continuously, where V_{th} increases if the specific neuron fires more than others and V_{th} decreases if the specific neuron fires less than others.

The full MNIST training database (60,000 digits) is processed by the system to achieve learning. Each input neuron is connected with one pixel of the image and fires pre-synaptic spikes (V_{pre}) that are proportional to the pixel intensity (the pulse timing of V_{pre} (t_{pre}) is proportional to the pixel intensity), as described in Ref. 26 and 27. The input neurons fire pre-synaptic spikes corresponding to a given digit within 50 ms, after which they fire spikes corresponding to another digit. No type of preprocessing is used on the digits, and the set is not augmented with distortions. The network is then tested on the MNIST test database, which consists of 10,000 digits that were not available to the system during training.

2. Electrical properties of CNT synaptic transistors and their variability

To date, two-terminal resistive switching devices (known as memristors) are most actively applied as a synaptic device. However, the sustainability of memristors is still in doubt, particularly regarding the uncontrollable weight change that is common to all memristor technologies. The specific physical mechanism of the conductance change in most prospective metal-oxide-based memristors, which is based on an atomic-scale modulation of oxygen vacancies based on an uncontrollable electro/thermo-dynamic feedback process, is responsible for the unwanted abrupt conductance change and consequent device-to-device variation. In addition, since most of memristors are based on an amorphous material, device-to-device variation caused by atomic-scale defect or irregularity is not intrinsically avoidable, which cannot be overcome by advanced fabrication technology.

We demonstrate CNT network transistors constructed from highly purified 99%-semiconducting CNT solutions processed using a density gradient ultracentrifuge separation method[29,30]. The removal of metallic CNTs *via* a solution process for the semiconducting CNT network has been shown to dramatically improve the electrical performances of CNT transistors, including a decrease in the leakage path, achieving a high I_{on}/I_{off}. Moreover, a high uniformity and high device yield could be achieved simultaneously.

Figure S2a shows the transfer characteristics for a total of 19 devices, which had a channel length of 1.9-3.0 μm and a channel width of 2.0-5.0 μm; measurements were made at room temperature and in ambient air. The as-fabricated devices initially exhibited p-type behavior, as demonstrated by the transfer characteristics (I_D-V_G; drain current-gate voltage) at a drain voltage (V_D) of -1 V. The device exhibited a peak on-current density (I_{ON}/W) of 24 μA/μm and a peak transconductance (g_m/W) of 6.85 μS/μm as normalized by the channel width. Other major

electrical parameters are also summarized in Figs. S1b-d. Excellent and uniform electrical performances were achieved in terms of the threshold voltage (V_T), the on/off current ratio (I_{ON}/I_{OFF}), and the mobility (μ) in the CNT transistors due to the highly purified, pre-separated 99%-semiconducting CNTs. In addition, the variabilities in the electrical performances in the CNT transistors were considerably lower than the variabilities in conventional two-terminal-based resistive devices (*i.e.*, conventional memristors), representing a significant advantage for implementation in large-scale synaptic transistor networks.

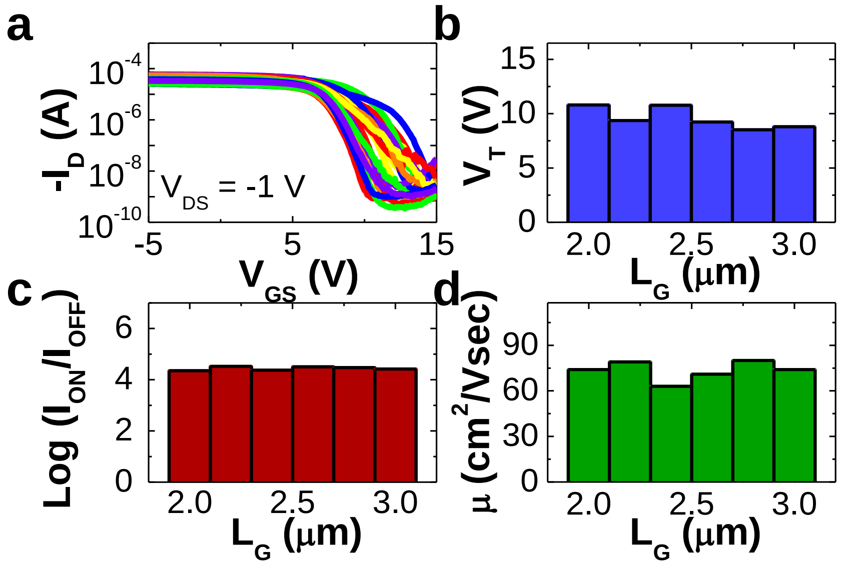

Figure S2. (a) Transfer characteristics (drain current, I_D versus gate voltage, V_G) for a total of 19 devices. (b-d) Distribution of the electrical performances of the fabricated CNT transistors with various channel lengths (L_G). Uniform and excellent electrical properties for different L_G were obtained.

3. Hysteresis and consequent channel conductance modulation in CNT synaptic transistors

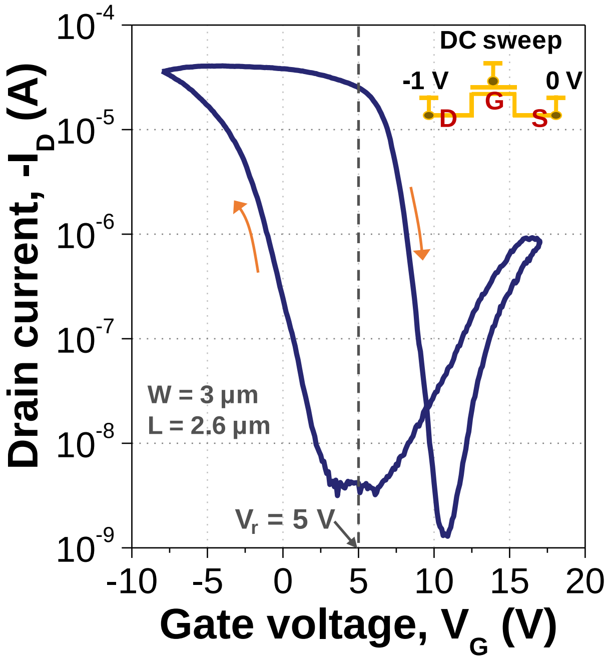

Figure S3. Hysteresis of the drain current (I_D) as a function of the gate voltage (V_G) with a constant drain voltage (V_D = -1 V).

The synaptic weight can be reproduced by an intrinsic analog state of the channel conductance in the CNT synaptic transistor. A hysteresis in the drain current (I_D) under a constant drain voltage (V_D) was observed when the gate voltage (V_G) was swept from -8 V to +17 V and back to -8 V (Fig. S3); a negative V_G increased the channel conductance, which is defined as the 'potentiation' of the synaptic weight, and a positive V_G decreased the conductance, which is defined as the 'depression' of the synaptic weight. This hysteresis indicates an intrinsic characteristic of the variable channel conductance in the CNT synaptic transistor that is dependent on the control of V_G.

The hysteresis in the CNT transistor stems from charge trapping at the Au floating-gate. When a negative V_G is applied, the electrons from the gate electrode tunnel and become trapped in the

Au floating gate, which leads to an increase in the channel conductance due to the decrease in the threshold voltage. In contrast, a positive V_G enables ejection of the trapped carriers at the Au floating gate, which results in a decrease in the channel conductance. This trapping/de-trapping effect of the carriers provides internal dynamics that drive the analog channel conductance switching behavior. Notably, this hysteresis of the channel conductance with long-term retention is an invaluable characteristic for the implementation of synaptic functions. Channel conductance can be modulated accurately in an analog manner by simply adjusting the amplitude of V_G (without any specially designed pulse shape or timing), which is of particular importance because it is not easily obtainable from other synaptic devices based on two-terminal resistive switches.
